# Is badfiction processed differently by the human brain? An electrophysical study on reading experience

**DOI:** 10.3389/fnhum.2023.1333965

**Published:** 2024-01-26

**Authors:** Thomas Weitin, Thomas Fabian, Anastasia Glawion, Judith Brottrager, Zsofia Pilz

**Affiliations:** ^1^LitLab, Darmstadt University of Technology, Darmstadt, Germany; ^2^Cognitive Psychology Unit, Faculty of Social and Behavioural Sciences, Leiden University, Leiden, Netherlands

**Keywords:** neurocognitive poetics, neuroaesthetics, neuroliterature, fanfiction, EEG, reading experiment, literature perception, cognitive humanities

## Abstract

Literary reception is a special case of language processing. The judgment of literature reveals deep social patterns with embodied cognition. In this study, we investigate how differences in literary quality resonate in the human brain. Modifying a series of stimuli previously used in studies of the emotional potential of *Harry Potter*, we alternate passages from the original novels with passages from imitative and intentionally poorly written fanfiction. EEG data shows how the three text types are processed differently by the brain. Comparing the brain activity of the readers for the various text types, we see a difference in the absolute power but not in the relative power of the frequency bands. Reading badfiction evokes the lowest activity. However, the functionality of this activity is the same for all texts since the relative power of the frequency bands does not differ. When comparing the participant groups, we observe the opposite situation. Here, different relative powers of the frequency bands reflect different judgments and reading habits of participants. For example, fans of *Harry Potter*, regular readers of fantasy texts, and generally frequent readers read the texts more attentively, which is reflected in a pronounced relative activity of the theta and alpha frequency bands. Non-frequent readers and readers who are not devoted to *Harry Potter* and fantasy in general have increased activity in the delta frequency band. This suggests their saliency detection is more prominent because they are less familiar with reading or the subject matter. To support our findings, we use the EEG data without averaging over stimuli and participants, capturing the participants' responses on the level of individual stimuli. A Kohonen self-organizing map trained on this more extensive data finds reliably detectable differences in the responses to passages from the original *Harry Potter* novels and fan- and badfiction. Our study allows for an interpretation of an adaptive brain response. Readers who enjoy *Harry Potter* or have experience with the fantasy genre show different reactions from those who do not. Thus, badfiction appears to be processed differently by the human brain, but not for all readers in the same way.

## 1 Introduction

Judging literature as good or bad is a matter of individual taste and, at the same time, a necessarily social act. Although nobody has ever been convinced of a novel's beauty by aesthetic reasoning (Kant, 2007), what we like and dislike reveals deep cultural patterns that might even be embodied in the brain (Berridge and Kringelbach, 2015; Morales and Berridge, 2020). A recent large-scale study in computational literary studies tried to identify textual features responsible for literary quality and found readers' judgment to be deeply biased instead (van Dalen-Oskam, 2023). Except for certain periods, such as Victorianism in the Anglophone and *Goethezeit* in Germanophone literary history, there is almost no evidence that the processes of agreement on what is good and worth becoming cultural heritage, known as canonization, can be predicted from the very structure of literary works (Brottrager et al., 2021). However, chances to predict the popularity of a work are slightly better (Archer and Jockers, 2016; Brottrager et al., 2022).

Since a fundamental concept such as the quality of literature cannot be explained using what most researchers believe is the primary method of philology, textual analysis on single work or corpus level, reception and reader-response are of growing importance again. Former booms of impact-oriented research during 19th-century positivism (Barsch, 2000; Kaltenbrunner, 2010), in early structuralism (Jakobson, 1960), and especially with the school of Constance (Holub, 2008) saw reader-response as a one-directional process caused or even implied (Iser, 1980) by textual structures. New developments in neurocognitive poetics (Jacobs, 2015) foster a view that considers the complexity of neural processing in the brain as a default condition of the responding reader.

Building on these theories of literary reception, we aim to examine readers' reactions to a very specific and highly conditional literary genre that is, based on this specificity and conditionality, prone to elicit strong reactions: Badfiction. A play of words on the broader genre of fanfiction, i.e., a literary practice that comprises texts that continue, supplement, or modify the story of an original work of fiction (Coppa, 2006; Tosenberger, 2014), purposely badly written badfictions challenge and play with readers' expectations and thus build on readers' existing reading experience. By comparing reactions to badfiction excerpts with passages taken from the original reference text and standard fanfictions in an exploratory analysis, we aim to contrast the influences of the presented stimuli with their specific textual characteristics on the one hand and the recipients and their reading experience and expectations on the other. Methodically, we combine a spectral analysis of absolute and relative Power Spectrum Density (PSD) values.

Numerous EEG studies have been conducted to investigate the issue of reading experience or, more broadly, general reading ability. Ackerman et al. (1998), for example, examine the neuronal activity of phonetic and dysphonetic readers, i.e., participants who are more or less adept at pronouncing nonsense words. They detect a higher beta power in dysphonetic readers when reading texts, from which they deduce a greater effort for dysphonetic readers. Galin et al. (1992) use an EEG to investigate how people with dyslexia read texts aloud or silently, showing that dyslexic readers do not differ from the control group in their EEG measurements. However, they find that the difference in activity in the theta band is significantly smaller in dyslexic readers than in the control group without dyslexia, indicating that dyslexics have more difficulties adapting their reading behavior to a new situation. Exploring the influence of the familial context on reading performance, Schiavone et al. (2014) investigate the reading behavior of children whose parents perform poorly on reading tests. For this, the children are divided into fluent and non-fluent readers on the basis of a reading assessment in the third grade. The special aspect of this study is that the eyes-open rest EEG measurements were carried out on the children at the age of three, i.e., even before they were able to read. The study indicates that future fluent readers already differ in their neural activity at the age of three, exhibiting lower activity in low-delta and higher activity in low-alpha frequencies. Thus, even before humans learn to read, they seem to have a predisposition to reading difficulties, which can be measured with an EEG.

Within the large field of reading research, empirical studies on literary reception can be considered special cases because literature, by definition, emerges through a particular use of language. The concept of literature itself, of course, varies over historical periods. However, since the emergence of modern authorship, there has been a coevolutional consensus that a literary voice needs to disguise its artificial language *naturally*. Any empirical investigation of the effects of literary texts faces the constraint of using stimuli from works with language variants that are not too dissimilar to today's readers' experience because otherwise, one would only measure the strangeness of historical variants (Schneider, 2000). This surely is the main reason why recent studies focus on contemporary literature with a recognizable emphasis on prevalent genres such as proverbs, game narratives, functional headings, or fantasy (Altmann et al., 2012; Bohrn et al., 2013; Hsu, 2015; Chen et al., 2023).

We have chosen badfiction as the subject of the current study because, due to its genre characteristics, it does not only lend itself well to our experimental set-up, comparing influences linked to texts and those related to readers, but because it is part of a highly relevant literary phenomenon. With the online fandoms of the internet era, these kinds of texts have reached a high proliferation and are today by far the fastest-growing part of contemporary literature (Coppa, 2017). Fanfiction communities, like other social media hot spots, follow certain communication habits whereby the first rule for fans is not to criticize each other's work too harshly (Evans et al., 2017; Kelley, 2021). Since leaving negative comments plays out badly in the community, publishing texts written poorly on purpose has become a trend that serves as a parody-like form of critique (Weitin et al., 2023). Badfiction texts can be characterized by the consequent undermining of literariness both in terms of general expectations about good writing and subject related conventions. This is accompanied by profanity, linguistic simplicity, unexplained turns of events, and figures acting out of character. In such a way, badfictions not only turn foes into friends or establish surprising relationships (which is characteristic for fanfiction in general), but employ literary means to narrate adaptations of reference material that are purposefully poor, yet linguistically correct (Table 1). The texts in question are intriguing to examine because their creators themselves have deemed them to be inferior within a tightly-knit literary community. Hence, what “bad” means is kept unbiased by outside judgment, academic or not.

**Table 1:** Examples of badfiction stimuli.

**German original**	**Translation**
Cedric wechselte seine Kleidung nicht mehr, wusch sich nicht mehr und verwahrloste. Aufgrund mangelnder Körperhygiene fing er an, ganz fürchterlich zu stinken, bis Cho ihn in den Schwarzen See schmiss, um ihn zu waschen. Aber auch das half nichts mehr und so war es eine große Erleichterung, als er endlich starb (Noctua, 2014).	Cedric stopped changing his clothes, stopped washing himself and fell into disrepair. Due to a lack of personal hygiene, he began to stink terribly until Cho threw him into the Black Lake to wash him. But that didn't help either, so it was a great relief when he finally died.
Und es zog ihn hin zu ihm mit Macht, und er spürte die Wärme seines Körpers und den herben Geruch nie gekannter Salben. Und sie sahen, dass es gut war. Bis Ron sich auf einem Arm hochstemmte und sagte: “Harry. Du stinkst nach ranzigem Schaf” (Liriaen, 2006).	And he was drawn to him with power, and he felt the warmth of his body and the tangy smell of unprecedented ointments. And they saw that it was good. Until Ron leaned up on one arm and said, “Harry. You reek of rancid sheep.”

It is an established practice to ask participants of a literary reading experiment to rank the stimuli they just read after scales of liking (Võ et al., 2009; Citron, 2012; Jacobs, 2015; Jacobs et al., 2016), aesthetic value (Leder et al., 2004; Leder and Nadal, 2014; Jacobs, 2015; Schindler et al., 2017), or receptional success in terms of immersion (Green, 2004; Hsu et al., 2014), absorption (Tellegen and Atkinson, 1974; Green and Brock, 2000), or emotional involvement (Andringa, 1996). Such rankings are also used in pre-studies to check which stimuli fit target variables best. However, to our best knowledge, no experiment has been conducted so far using self-declared badfiction. When we investigated if the human brain would process badfiction differently, we selected examples from a *Harry Potter* subsection on a fanfiction forum. We compared them with regular fanfiction on *Harry Potter* from the same forum and with stimuli from the original novels previously used in emotional potential studies (Hsu, 2015; Jacobs, 2019).

Being historically the first experimental technique to provide evidence for the concept of functional brain localization (Aron et al., 2021), today, the use of electroencephalography (EEG) is still on the rise due to its ability to characterize brain functioning (Fabio et al., 2016) whereby the communication between different brain areas has become of growing importance (Stam and van Straaten, 2012) which is true for fMRI studies as well (Price, 2012). With quantitative electroencephalography (QEEG), key parameters like the power spectrum of the frequency bands and their interpretable functionality have been consolidated and proven to be a proper means for diagnosis and treatment of mental diseases such as ADHD (Pop-Jordanova and Pop-Jordanov, 2005; Lenartowicz and Loo, 2014; Anchana and Biju, 2023), Alzheimer's disease (Cassani et al., 2018; Rossini et al., 2020; Vicchietti et al., 2023), autism (Billeci et al., 2013; Wang et al., 2013; Ribeiro and da Silva Filho, 2023), and epilepsy (Acharya et al., 2013; Tatum et al., 2018; Aron et al., 2021).

That QEEG can be useful for the study of literary reception seems plausible given its worth for the analysis of language functions (Dimigen et al., 2011; Gaudet et al., 2020; Zhou et al., 2020), the reception of fine art (Umilta' et al., 2012) and the recommendation of this method in connection with the neurocognitive poetics model (NCPM) (Jacobs, 2015). Given the different effect sizes between clinical studies on severe brain alterations and a reading experiment that tries to measure neural correlates of the comparatively fine distinctions of literary quality, applying QEEG to philology needs particular caution and scrutiny. If Bourdieu (1984) is correct, and it is indeed the small differences that make up for cultural distinctions in the taste for art, brain reactions to variations of a literary work should differ only slightly.

As we expect small but significant differences in brain reactions to our three types of literary stimuli, a method like Kohonen Self-Organizing Maps (SOM) seems appropriate to consolidate the results. The SOM, which is a type of artificial neural network (ANN), detects similarities between the responses on a stimulus level and organizes them into clusters of similarity. These clusters can be used to predict which type of text (badfiction, fanfiction, or original) a participant is reading based on their brain activity.

We assumed that badfiction would make a difference because of its ability to disturb what readers expect and what they are familiar with. Previous research on popular literary genres has found evidence that familiarity causes stronger brain effects than liking (Bohrn et al., 2013). We tend to like what we know. If this is true, the disturbance of the familiar, which, according to the NCPM, forces the reader into the poetic mode where “unusual” (Jacobs, 2015, p. 16) elements are encountered, should show some effect. This was the idea when we set up trials where passages from the original *Harry Potter* novels were alternated with fanfictions that are, in principle, in line with the original universe and badfictions that turn it upside down (see Section 2 for details).

Per its definition, fanfiction requires being familiar with the original work it is about, in our case *Harry Potter*. Moreover, badfiction, as a subgenre, certainly needs an even higher level of familiarity to fulfill its critical ambitions, purposefully disappointing other readers' expectations. Hence, when we want to analyze how differences in literary quality, specifically between original fiction, fanfiction, and badfiction, would resonate in the human brain, we need to consider familiarity as a gradual phenomenon. This is with respect to previous research that highlighted how experienced and unexperienced readers differ in their dependency from context knowledge (Benau et al., 2011). As inexperienced readers rely more on context, they are more likely to struggle when incongruencies in the literary narration occur. We decided to measure familiarity with post-experimental questionnaires and evaluation tasks starting on the general level of familiarity with the literary system and reading habits in terms of reading frequency in everyday life down to genre-related preferences and the attitude toward badfiction stimuli used during the experiment.

As this work aims to compare the influence of object and recipient, there are two focus areas we are especially interested in. On the one hand, we investigate the intersubjective influence of text type variation on readers. On the other hand, we consider the neural response of individual groups of readers with shared personal preferences and reading habits. Through this comparison, we hope to find discrepancies in the influence of text and reader variation. We hypothesize that badfiction is processed differently by the human brain, but not for all readers in the same way. Badfiction in itself should influence the mental effort required by the reader because of its simple language and structure. We believe the influence on the functionality of brain activity should not depend on the text but on the reader, whose specific habituation yields subjective differences.

## 2 Methods

### 2.1 Material

As mentioned above, a main characteristic of badfiction texts is that they are subverting literariness by acting against readers' broader presumptions about a well-written literary text but also the rules and regulations inherent in the main genre of fanfiction. Consequently, even though badfiction texts are grammatically, syntagmatically, and orthographically correct, they are deliberately written to be “bad” fiction. We used 15 badfictions with a length of approximately 40 words from the *Harry Potter* fandom of the Germanophone web forum fanfiktion.de. These short *Harry Potter* narratives had been marked as “badfictions” by their authors. To compare them with conventional fanfiction, we again chose 15 texts of approximately 40 words from the same fandom that bore no specific genre description but were labeled as “general” (German: “allgemein”). For the comparison with the original *Harry Potter* novels, we used 120 text excerpts of approximately 40 words from a previous study on emotion potential (Hsu, 2015) that had worked with a German translation. In advance, we conducted an anonymous online pre-study with 82 participants (64 female, 6 male, 5 non-binary, 7 not specified). Twelve participants were younger than 18, 47 were between 18 and 29, 21 were above 29, and two did not specify their age. All participants in the pre-study were native German speakers or near-native German speakers. We obtained informed consent from all participants. By performing the pre-study, we ensured that conventional fanfiction and badfiction stimuli were sufficiently different. For that purpose, a five-tier Self-Assessment Manikin (SAM) scale was presented to measure arousal, valence, and dominance. Fanfictions and badfictions for the main experiment were chosen to maximize the difference between the two groups concerning these scores. Badfictions had particularly high valence (positive emotion) and low dominance; fanfictions had low valence (negative emotion) and high dominance. Given previous research that has shown how grammatical errors influence neural processing (Schneider et al., 2016), for all fanfiction and badfiction stimuli, orthographic and grammatical errors have been corrected. By doing so we wanted to make sure that the experiment measures only the intended irritations typical especially for badfiction.

### 2.2 Participants

In sum, 40 participants (20 female, 18 male, two non-binary) took part in the study, all German native speakers. Recruited from TU Darmstadt, most participants (36) were between 18 and 29 years old (three 30–39 years old, one 60 or older). All participants had normal or corrected-to-normal vision and no self-reported reading impairments. The experiment was approved by the Ethics Committee of TU Darmstadt and complies with the Declaration of Helsinki.

### 2.3 Task

During the experiment, the 40 participants read texts from all three *Harry Potter*-related text types while their brain activities were measured via EEG. Stimulus presentation was divided into 15 trials. In each trial, a test subject had to read four texts from the original *Harry Potter* (translated into German) followed by one conventional fanfiction. Then again, four originals were presented, followed by one badfiction. Stimuli of the three text types were randomized within their group. Thus, over the 15 trials, each participant read 150 stimuli consisting of 120 originals, 15 fanfictions, and 15 badfictions. After every sixth stimulus, an attention question was included. To answer, participants used a computer mouse. For stimulus continuation, they used a button box.

After the measured reading had ended and the electrodes were removed, the subsequent survey part started with an evaluation task focused on badfiction. Two randomly chosen badfiction stimuli already presented in the experiment were presented again, each with the task for participants to describe their impression in three to five keywords. Afterward, participants filled out three questionnaires, the first to check familiarity with the fictional world of *Harry Potter*. In the second questionnaire, participants provided information on to which extent they had consumed *Harry Potter* novels, movies, and additional material. This was done with a questionnaire developed by Kuijpers et al. (2020) that allows for a genre-specific acquisition of reading frequency. Lastly, participants took an author-recognition test (Grolig et al., 2020), where they had to choose authors they were familiar with from a list of 75 authors' names. Since the list is a mixture of real and made-up names, this test is supposed to capture familiarity with the literary system in general.

### 2.4 Data acquisition

The participants were seated in a soundproofed, dimly lit booth, shielded from natural light. They sat at a distance of about 85 cm from a 60 × 34 cm screen (Lenovo ThinkVision, resolution of 1280 × 720 pixel). The stimuli occupied the entire screen, giving them a visual angle of about 32°. The texts consisted of black letters on a gray background with moderate luminance. Participants were told to move as little as possible so as not to contaminate the data. Reading all stimuli required around 1−1.5 h. The subsequent completion of the questionnaires took about 5−10 min. Together with attaching the electrodes, removing the electrodes and participants washing out their hair, the whole experiment took around 2−2.5 h.

EEG activity was recorded using a setup with 32 shielded Ag/AgCl electrodes in *acticaps* (Brain Vision Solutions Inc., Canada) with a ground electrode at the forehead and a reference electrode placed at a central position on the skull between Fz and Cz electrodes. Following the international 10/10 system, we placed the electrodes at the positions Fp1, Fp2, Fz, F3, F4, F7, F8, FC1, FC2, FC5, FC6, FT9, FT10, Cz, C3, C4, T7, T8, CP1, CP2, CP5, CP6, TP9, TP10, Pz, P3, P4, P7, P8, Oz, O1, and O2. An electrolyte gel was used to keep the impedance between electrodes and skin below 20 kΩ. We used an MES LiveAmp wireless system (Brain Products GmbH, Germany) to amplify the signals from the electrodes. To increase the recording quality, we aimed for a high input and differential impedance (Guo, 2020). The used input impedance was >200 MΩ (at direct current) of the EEG channels to the ground electrode. The differential input impedance between two electrodes was >400 MΩ to minimize ambient recording. The EEG data were digitized at a frequency of 500 Hz. The EEG recording in each trial started when the text appeared and ended as soon as the participant confirmed reading the text by pressing a key. Between two trials, a drift check was carried out during which no EEG data was recorded (drift checks were carried out for eye tracking, but we do not use its data here). The next trial started immediately after the drift check. For data cleaning and analysis, EEGLAB, an EEG extension for MATLAB (Delorme and Makeig, 2004), was employed.^1^

### 2.5 Data preparation

Since the collected EEG data contained much noise in the low-frequency range, we needed a sharp cut-off to eliminate it. Therefore, the decision for an infinite impulse response filter (IIR) was made. For the high-pass, we set the filter with order six and a transition bandwidth of 0.2 Hz at 0.5 Hz. This means that below a frequency of 0.5 Hz, signal reduction begins. Down to the frequency of 0.3 Hz, the signal is reduced in a steep filtering process. At the latest below 0.3 Hz, it should be at zero (due to the stopband attenuation, the value is only approximated—for a complete theoretical explanation of all filtering details, see Widmann et al., 2015). At the upper end of the passband at 40 Hz, we applied the filter with order twelve and a transition bandwidth of 1 Hz. Lengthening the transition bandwidth results in less distorted data. At the upper end, a shallower filter is affordable since there is less noise than in the low-frequency range. Hence, the filtering starts above a frequency of 40 Hz, and from 41 Hz, the signal is approximately at zero. We applied the two IIR filters to each data set, i.e., 150 times for the three text sort conditions of all 40 participants. With both filters, mainly the low-frequency noise below 0.5 Hz was removed, but also the frequencies above 40 Hz that are beyond our interest.

Channels were manually inspected by two researchers who sorted out erroneous and too noisy channels (Cohen, 2014, p. 80–81). We decided to handle artifact rejection similarly because this enabled us to address interpersonal differences. An Independent Component Analysis (ICA) (Onton et al., 2006; Delorme et al., 2007; Debener et al., 2010) was employed to detect muscle noise and eye movements. EEGLAB allows automatically labeling components with an estimate of the probability of this prediction being correct. We generally removed components from the data classified as muscle artifacts or eye movements with a probability of at least 85%. For noisier data, especially when the ICA had trouble identifying any components, we rejected components with only 80 or 75% certainty of being an artifact. We ensured the artifacts were correctly removed by a final manual examination of the data.

### 2.6 Data analysis

In preparation for spectral analysis, the continuous data was segmented into individual epochs of 1 s. We used overlapping Hamming windows to prevent the creation of new artifacts due to subdivision. With a repetition rate of 0.5 s, we gained a window overlap of 50%, providing enough space for artifact removal but keeping the operational effort at a bearable level. A fast Fourier transformation (FFT) was then used to transfer the activities from the time domain into the frequency domain (Nussbaumer, 1981; Brigham, 1988). Once all segments had been double averaged over text sort and participants, a 1-way ANOVA was performed to find areas where our three textual reading conditions—original fiction, fanfiction, and badfiction (all about *Harry Potter*)—differ significantly. We chose permutation statistics since we could not assume an underlying, well-defined distribution in our data but had enough data at hand. We used the Bonferroni correction with a significance threshold of *p* = 0.05 to correct for multiple comparisons.

To consolidate the findings of the spectral analysis and examine the EEG data on a different level of granularity without averaging, we implemented a Kohonen self-organizing map (SOM). Using the more extensive data set of all individual participants' responses on the level of the individual stimulus, the SOM, which is a type of artificial neural network (ANN), detects similarities between the responses on a stimulus level and organizes them into clusters of similarity. These clusters can be used to predict which type of text (badfiction, fanfiction, or original) a participant is reading based on their brain activity. Since the Kohonen map implementation used (Wehrens and Buydens, 2007; Wehrens and Kruisselbrink, 2018) requires complete data, six electrodes that had not correctly recorded for some of the participants were removed. This left us with 1,350 stimuli to be divided after a 70/30 ratio for training and test data. To avoid distortion by individual patterns, all stimuli of a single participant were used either for training or testing. During the experiment, every trial consisted of four *Harry Potter* originals, one fanfiction, four originals again, and one badfiction. For SOM training and testing, the three text sorts were used proportionally.

The analysis of relative powers of frequency bands was also computed on the extensive, stimulus-based data set. Our focus here was on the influence of reading habits and different levels of familiarity with *Harry Potter*, the fantasy genre, and the literary system in general. From our metadata, five variables were selected to build groups for statistical analysis: reading frequency (frequent vs. non-frequent), familiarity with the literary system according to the author-recognition test (buffs vs. rookies), fantasy reading according to the genre-specific reading habit questionnaire (fantasy reader vs. non-fantasy reader—participants who had read at least one text of the genre in the last 12 months were classified as fantasy readers), familiarity with *Harry Potter* (fan vs. non-fan—participants who consumed all novels and movies were considered as fans), attitude toward badfiction (positive vs. negative—manually classified based on the keywords participants used in the post-experimental evaluation task). Whereas, “fans vs. non-fans” and “fantasy vs. non-fantasy” were binary distinctions, the other variables entailed rest groups which we neglected for the analysis (see Supplementary material for details).

For 31 channels (Fp2 electrode had to be excluded due to poor data quality), five frequency bands and the brain rate (Pop-Jordanova and Pop-Jordanov, 2005) were tested to examine if the five variables bear significant differences along the binary groups. Hence, 31 × 6 × 5 = 930 tests had to be conducted. For every pair of groups, we tested the homogeneity of variances with Levene's test. With a Shapiro-Wilk test, we checked for normal distribution. If both conditions were fulfilled, a *t*-test for independent samples was performed. In case of no homogeneity of variances, we conducted a Welch's *t*-test. If the normal distribution was not given for at least one of the groups, a Mann–Whitney *U*-test was used, which was also applied if both conditions failed. To adjust the alpha level of *p* = 0.05, we used the False Discovery Rate (FDR) according to the Benjamini–Hochberg procedure (Benjamini and Hochberg, 1995). The adjusted alpha level is 0.01. In addition to statistical significance, we use Cohen's *d* (Cohen, 1969) to determine the effect size as a measure of practical significance.

## 3 Results

### 3.1 Absolute PSD values

**Figure 1:**
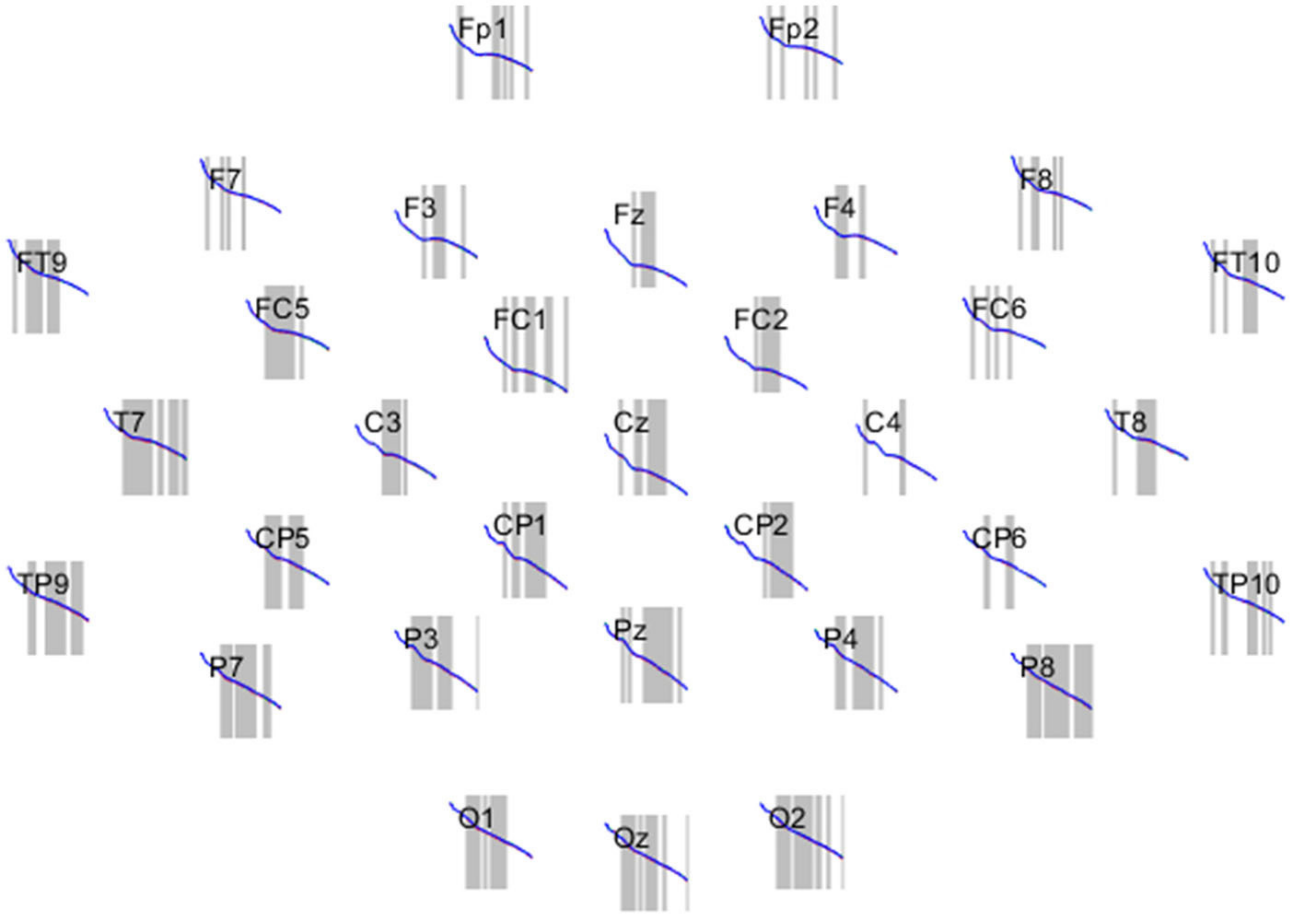
Power in the frequency spectrum from 0 to 40 Hz for 32 electrodes. All participants for three text sorts: badfiction (red), fanfiction (green), and original *Harry Potter* (blue). The curves are hardly distinguishable visually. Significant areas are marked gray.

We compared the absolute Power Spectrum Density (PSD) (Billeci et al., 2013) in the EEG frequency spectrum as a function of the independent variable text sort under the three experimental conditions: original *Harry Potter* fiction, fanfiction, and badfiction. As expected, differences between brain reactions to variations of a literary work are small but significant (Figure 1).

Furthermore, those differences were greater than those caused by the five metadata variables that helped us capture reading habits and different levels of familiarity with literature in general and the literary material we tested in particular. When we sorted the data after groups such as frequent and non-frequent readers, buffs and rookies of the literary system, or fans and non-fans of *Harry Potter*, the text sort differences between original fiction, fanfiction, and badfiction stayed more or less the same and significant. The other way around, with a division after text sort, almost no significant differences between metadata groups were found.

**Figure 2:**
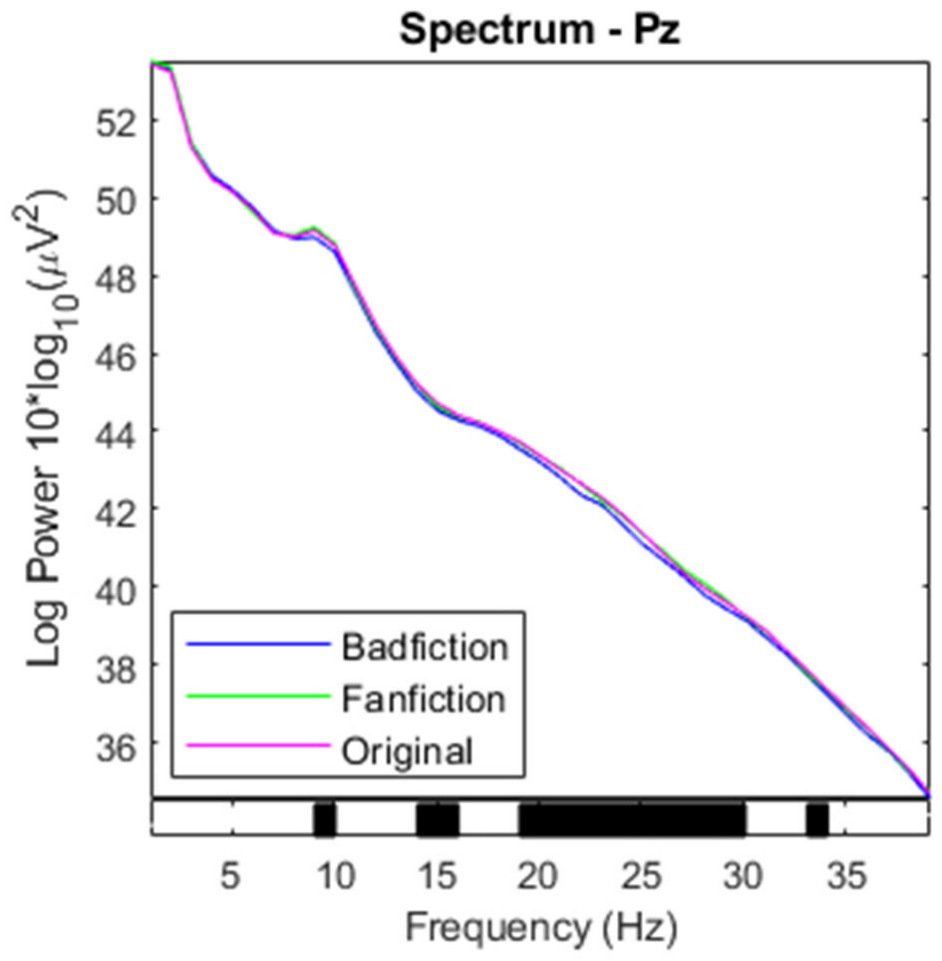
Power in the frequency spectrum from 0 to 40 Hz for Pz electrode. Black markers on the x-axis indicate significance.

We found that for all participants, absolute PSD was the lowest for processing badfiction, which can be seen in the capture of the central Pz electrode (Figure 2).

As badfiction uses simple language and lacks coherent narration, maintenance of the mental state and knowledge integration need fewer mental resources. However, we leave the interpretation for the relative PSD values that are more suitable to understand the activities of single frequency bands compared to the overall activity (see Section 4).

Given the small but significant differences in brain reactions to our three types of literary stimuli, a method like Kohonen Self-Organizing Maps (SOM) seems appropriate to consolidate the results. As for competitive learning with neural networks in Kohonen maps, each neuron adjusts its weight to match the incoming signal, whereby here, neighboring neurons are adjusted as well (Kohonen, 1990). That is why SOMs are often used for clustering. From a total of 1,350 stimuli, 945 were used for training and 405 for testing. The model we report here used 10,000 iterations on a hexagonal grid of 6 × 6 nodes and the default radius of the neighborhood (other configurations lead to similar results).

**Figure 3:**
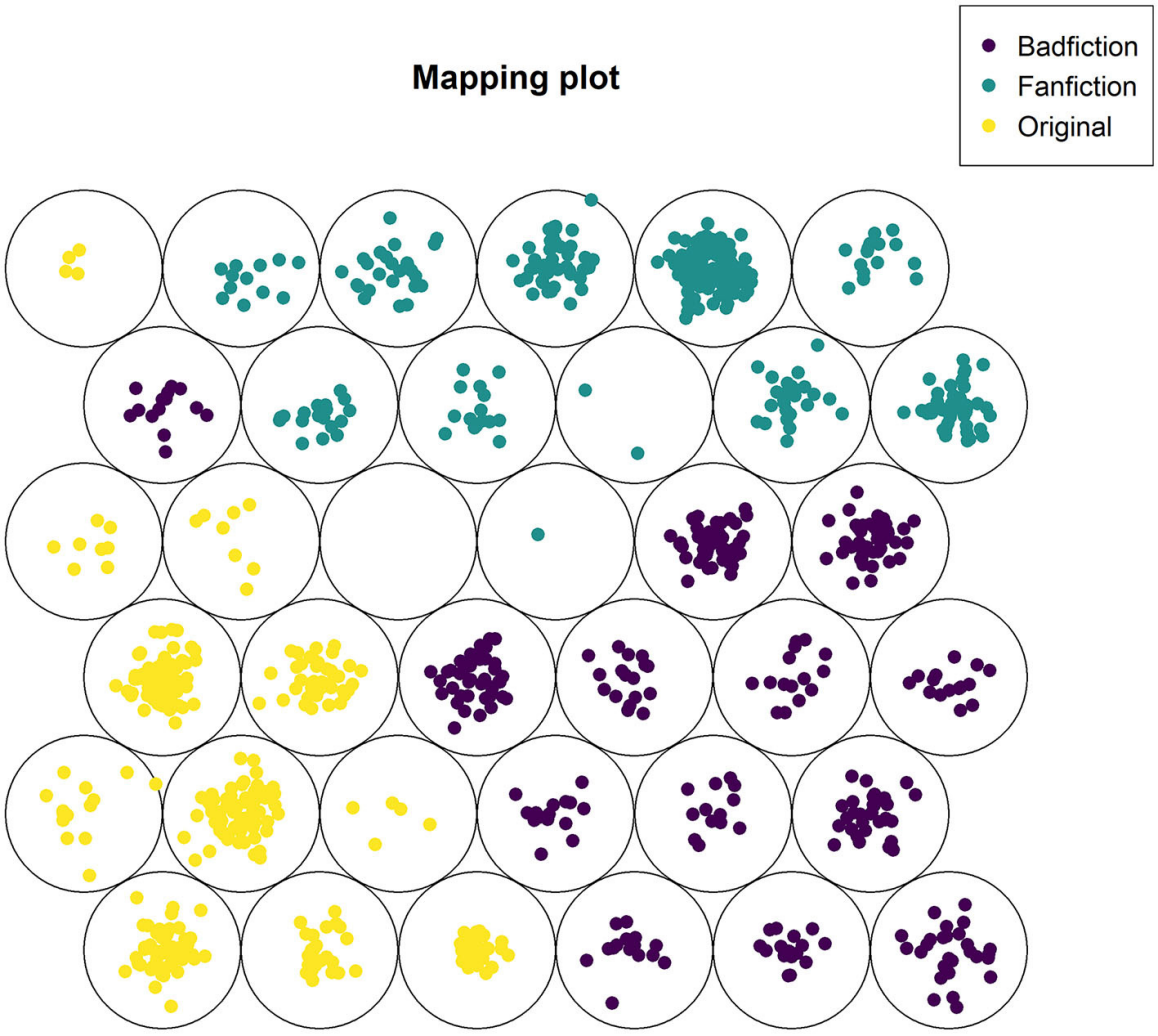
Kohonen Self-Organizing Map of PSD data for badfiction, fanfiction, and original fiction on *Harry Potter*.

The clustering of responses to original, fanfiction, and badfiction stimuli proved to be highly accurate (Figure 3), with only one badfiction stimulus being misclassified as an original (Table 2). Accordingly, the F1 score for badfiction and the *Harry Potter* originals is 0.996, while it reaches 1.00 for fanfiction. However, a mapping of metadata on the trained clusters that were consolidated by testing revealed that, at least to some extent, the strong grouping effect was caused not only by text sorts but also by different reading habits, various levels of familiarity with literature and its genres, and patterns in the data of individual participants. With these caveats in mind, the respective metadata groups needed further inspection.

**Table 2:** Confusion matrix test data SOM.

**Category**	**Predicted**			**Total**
	**Badfiction**	**Fanfiction**	**Original**	
Badfiction	134	0	1	135
Fanfiction	0	135	0	135
Original	0	0	135	135
Total	134	135	136	

**Figure 4:**
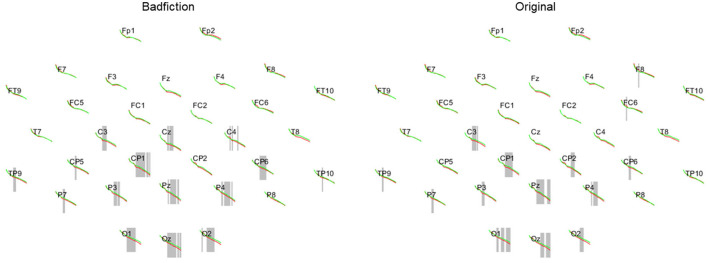
Power in the frequency spectrum from 0 to 40 Hz for 32 electrodes. Fantasy (red) and Non-Fantasy-Reader (green) for badfiction stimuli **(left)** and original *Harry Potter* stimuli **(right)**. Significant areas are marked gray.

As a first finding in that direction, we report discrepancies between two groups of readers that, according to the reading habit questionnaire, could be considered as either fantasy or non-fantasy readers. Figure 4 shows how the two groups differ significantly when they read badfiction on *Harry Potter* or text parts from the original novels. Those differences are located in the occipital region where visual processing and imagination take place (von Stein et al., 1993), and it is within this region that we see that for both reading conditions, fantasy readers (red) have a lower activity than their non-fantasy counterparts. This finding may result from greater familiarity with the fantasy genre, which presumably helps readers with genre-specific experiences to spare effort and become more resilient against the textual signal. Since we did not observe such a difference between neither frequent and non-frequent readers nor between different classes of familiarity with the literary system (buffs vs. rookies), the reading experience that matters here seems to be genre-specific.

### 3.2 Relative PSD values

Relative PSD is calculated as the ratio of a single band's mean absolute power and the spectrum's total absolute power (Billeci et al., 2013). We report the results for all 40 × 150 stimuli of the experiment related to our five metadata variables: reading frequency [frequent (*N* = 10) vs. non-frequent (*N* = 10) readers], familiarity with the literary system [buffs (*N* = 12) vs. rookies (*N* = 14)], fantasy reading [fantasy (*N* = 26) vs. non-fantasy readers (*N* = 14)], familiarity with *Harry Potter* [fan (*N* = 17) vs. non-fan (*N* = 23)], and attitude toward badfiction [positive (*N* = 9) vs. negative (*N* = 8)]. In the following, we refer only to significant differences between groups (for a complete list of means, standard deviations, *p*-values, and effect sizes, see Supplementary material).

Note that some of these groups have small sample sizes. Therefore, the results are subject to a non-negligible variance, meaning that we cannot claim general validity for the individual group comparisons. With these comparisons, we primarily want to investigate whether habitual differences between readers can be measured with an EEG. In addition, these comparisons serve as an initial point of reference for further studies with larger groups, which can validate the correlations we find in our rather small groups.

**Figure 5:**
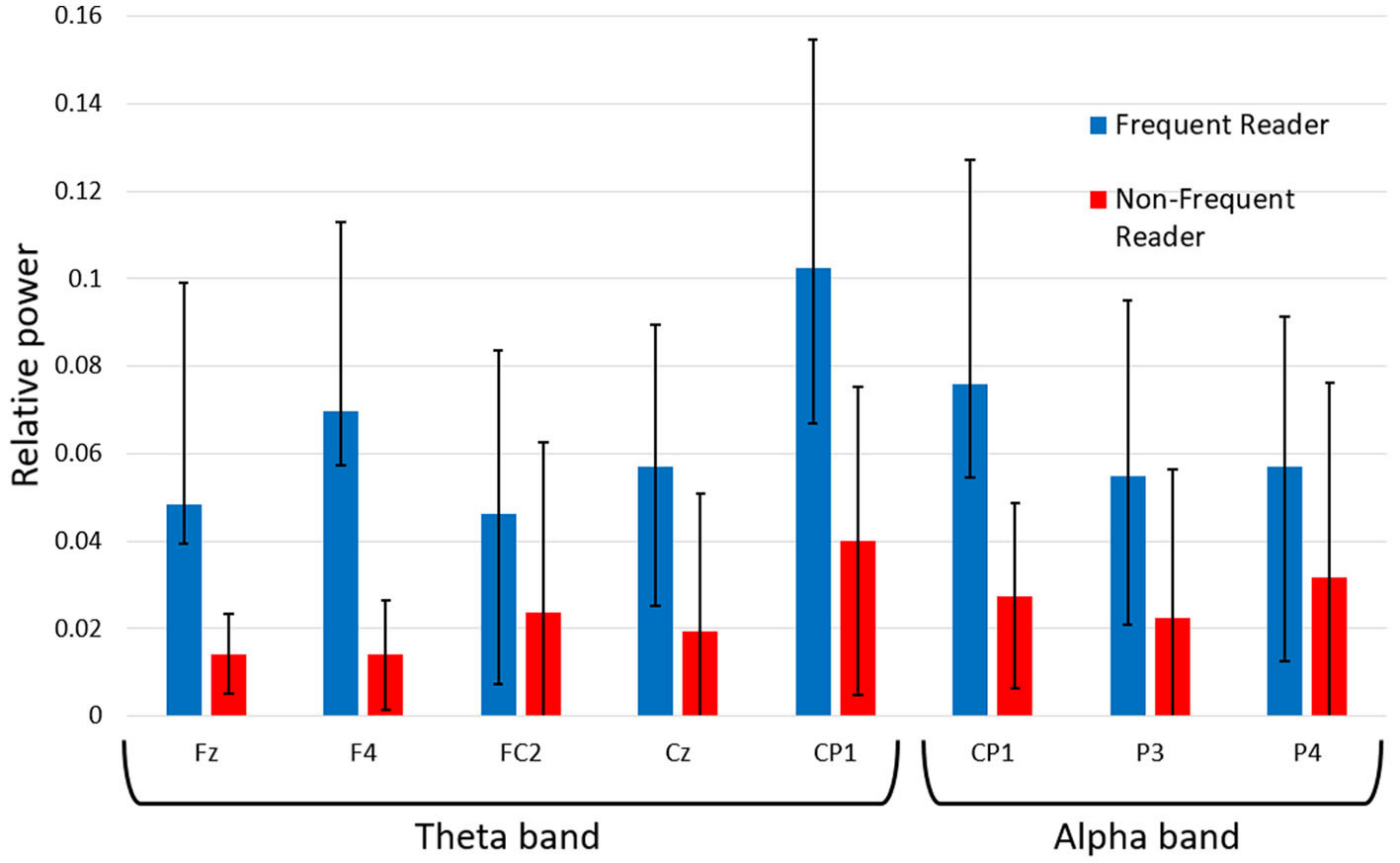
Means and standard deviations of the comparison of relative powers in readers reading all three types of *Harry Potter* stimuli.

Our results are shown in Table 3. We find increased delta power for non-frequent readers, non-fantasy readers, and non-fans compared to their respective complementary groups. Frequent readers (Figure 5) and fantasy readers show stronger activity in the theta band than their respective complementary groups. This difference is particularly evident in the anterior cingulate cortex. In the parietocentral regions, frequent readers and fantasy readers exhibit higher alpha band activity than their counterparts. Between fans and non-fans, the significant differences in the theta and alpha band are predominantly situated in the posterior and bilateral parietal regions. Moreover, fans show higher beta and gamma activity than non-fans. For readers with a negative attitude toward badfiction, we observe higher gamma activity than for positively attuned readers.

**Table 3:** Statistically significant results for the frequency bands.

**Delta**								
**Non-frequent**			**Non-fantasy**			**Non-fans**		
**Site**	***p*** **-value**	***d***	**Site**	***p*** **-value**	***d***	**Site**	***p*** **-value**	***d***
CP1	<0.0001	1.33	CP1	<0.01	0.85	C3	<0.01	0.17
Cz	<0.00001	0.97	FC5	<0.0001	0.75	CP6	<0.00001	0.78
F4	<0.0001	1.60	FT9	<0.01	0.42	F4	<0.000001	1.12
F7	<0.001	0.70	P4	<0.001	0.55	Oz	<0.00001	0.93
FC5	<0.001	1.00	P8	<0.01	0.75	P3	<0.01	0.63
Fz	<0.001	0.86				P4	<0.01	0.23
P3	<0.01	0.77				P7	<0.000001	0.75
P4	<0.001	0.68				P8	<0.001	0.72
**Theta**								
**Frequent**			**Fantasy**			**Fans**		
**Site**	***p*** **-value**	***d***	**Site**	***p*** **-value**	***d***	**Site**	***p*** **-value**	***d***
Cz	<0.00001	1.15	F3	<0.01	0.36	CP6	<0.00001	0.83
F4	<0.000001	1.72	F4	<0.01	0.51	Oz	<0.00001	0.90
F7	<0.001	0.72	F8	<0.01	0.53	P3	<0.01	0.56
FC2	<0.01	0.58	FC5	<0.0001	0.76	P4	<0.01	0.16
FC5	<0.001	1.06	Fz	<0.01	0.33	P7	<0.000001	0.75
Fz	<0.001	0.93				P8	<0.001	0.67
						TP10	<0.000001	1.14
**Alpha**								
**Frequent**			**Fantasy**			**Fans**		
**Site**	***p*** **-value**	***d***	**Site**	***p*** **-value**	***d***	**Site**	***p*** **-value**	***d***
CP1	<0.0001	1.21	CP1	<0.01	0.84	CP6	<0.0001	0.64
Cz	<0.00001	0.71	FC5	<0.00001	0.78	Oz	<0.0001	0.78
FC2	<0.01	0.60	FT10	<0.01	0.46	P3	<0.001	0.64
FC5	<0.001	1.15	P4	<0.0001	0.66	P4	<0.01	0.26
Fz	<0.01	0.81	P8	<0.01	0.75	P7	<0.000001	0.54
P3	<0.01	0.86	TP10	<0.001	0.71	P8	<0.001	0.69
P4	<0.001	0.62				TP10	<0.00001	0.96
**Beta**			**Gamma**					
**Fans**			**Fans**			**Negative**		
**Site**	***p*** **-value**	***d***	**Site**	***p*** **-value**	***d***	**Site**	***p*** **-value**	***d***
CP6	<0.00001	0.83	F4	<0.001	0.79	C4	<0.01	1.13
F4	<0.00001	0.98	FC5	<0.01	0.63	F7	<0.01	0.82
FC5	<0.01	0.56	FC6	<0.01	0.57	FC2	<0.01	1.31
Oz	<0.0001	0.94	FT9	<0.001	0.56	O1	<0.01	1.03
P3	<0.001	0.61	FT10	<0.01	0.54	T7	<0.00001	3.12
P4	<0.01	0.36	Oz	<0.0001	0.73	T8	<0.001	1.78
P7	<0.0001	0.69	P7	<0.001	0.48	TP10	<0.01	1.30
P8	<0.001	0.70	TP10	<0.0001	0.67			

Mentioned groups show more activity than their complementary groups.

## 4 Discussion

### 4.1 Absolute PSD values

As the results presented in Section 3.1 show, participants activity in the beta and low-gamma range is significantly lower when participants read badfiction than when they read excerpts from the original novels or normal fanfictions. We can explain differences for these frequencies since beta is associated with the maintenance of the current cognitive state (Engel and Fries, 2010), and gamma is related to the attentive reception of information and the integration of new information in the existing knowledge (Tallon-Baudry and Bertrand, 1999; Fries et al., 2001; Bauer et al., 2007; Jia and Kohn, 2011). Due to the simpler structure and limited plot of badfiction (see Table 1 for examples), reading these texts requires less active participation from the participants.

Furthermore, closer inspection of absolute PSD values reveals that fantasy and non-fantasy readers differ mainly in their beta band activity. According to previous research, this could mean that the non-fantasy readers' lack of experience of and exposition to fantasy themes and tropes needs to be compensated with more effort in preprocessing and mental structuring of the stimuli (Giannitrapani, 1971). Imagining unrealistic situations, such as the use of spells, seems to be a harder mental task for readers less familiar with these concepts. In contrast to this, fantasy readers already have mental structures available to process the presented stimuli, leading to lower beta band activity.

Besides the above described differences between fantasy and non-fantasty readers, we can find almost no differences between the different groups of readers using absolute powers of brain activity. However, if we compare different text types, badfiction elicits the least neural activity regardless of readers' preferences and reading habits. This finding confirms that the purposefully simple language and structure of badfiction influence readers' mental activity in an empirically measurable way. The weak but significant influence of badfiction shows that reading these texts requires less attentive participation on the readers' part, regardless of whether they like the text or frequently engage with such literature. Using absolute powers leads us to determine the intersubjective effort of reading, which is lower for badfiction for all readers.

### 4.2 Relative PSD values

Even though we have found some differences between reading habits that can be detected on the level of absolute values, to elaborate on differences between the recipients, we have to move to the relative values. By dividing the participants into habitual subgroups, some of the compared groups are quite small. Due to the small group sizes, we cannot claim general validity for these results. However, relative powers show different results than absolute powers, which reveals a conceptual difference between the two measures. For statistically more stable results, studies with more participants must be conducted.

We find higher delta activity for non-frequent readers, non-fantasy readers, and non-fans that could point to more prominent salience detection (Knyazev, 2007). This can be explained by the fact that all three groups have, for different reasons, a higher probability of encountering something unknown in their reading of *Harry Potter* variations. This would be consistent with the observation that no delta band power differences could be detected between readers with a positive or negative attitude toward badfiction.

A further influence of readers' habits on the cognitive states can be observed in the higher theta band activity that separates frequent and fantasy readers from their counterparts. This difference is particularly evident in the anterior cingulate cortex. Higher theta band activity in this area could be seen as a sign of increased memory load (Gevins et al., 1997). Frequent and fantasy readers, experienced and with affinity to the genre *Harry Potter* belongs to, seem to read the text more actively and therefore try to keep it in mind. Higher alpha band activity for frequent readers and fantasy readers in the parietocentral regions could be interpreted as a sign of skill development that allows experienced readers to proceed with fewer cortical resources (Gevins et al., 1997).

We also observe significant differences in the theta and alpha activity between fans and non-fans, although in different regions than for frequent and fantasy readers. Here, the distinctions predominantly show in the posterior and bilateral parietal regions. As for the alpha band, higher activity in this area hints at more items being processed in working memory (Jensen et al., 2002). Theta activity, in general, influences as a “navigation rhythm” (Buzsaki, 2005, p. 827) the formation of episodic and semantic memory. Higher beta power for fans can be explained with their context knowledge. As fans, they are highly familiar with the narrative *Harry Potter* world and can anticipate what could happen next. Therefore, it can be assumed that fan-readers maintain their current cognitive state during reading rather than expect changes, characteristic of strong beta band activity (Engel and Fries, 2010).

In addition to these observations for frequent readers, fantasy readers and fans, we also find a significant difference for participants with a negative attitude toward bad fiction. We see higher gamma band activity compared to those with a positive attitude. Given that gamma oscillations are associated with emotional memories and can be enhanced through emotionally aversive stimuli (Headley and Pare, 2013), the result for readers with an aversion against badfiction is explainable. Although the attitude measured in the evaluation task concerned only badfiction stimuli (15 of 150), it is strong enough to make a difference for the whole experiment.

Apart from these straightforward findings, we also make an observation that requires a more detailed interpretation. Readers with positive (*N* = 9) or negative (*N* = 8) attitudes toward badfiction build the smallest of our metadata groups. In contrast, the difference between fans (*N* = 17) and non-fans (*N* = 23) maps the total of all participants. Both groups overlap strongly, though, because of the difference in size, not symmetrically. Seven out of the eight readers who dislike badfiction are non-fans. At first glance, it comes as a surprise, then, that fans instead of non-fans show higher gamma activity. We explain this with the fact that the attitude toward badfiction captures extreme subgroups of fans and non-fans. Without the middle ground, the gamma power results turn upside down. The middle ground comprises the majority of readers who are ambivalent toward badfiction. Within this ambivalent majority, obviously, *Harry Potter* fans show a higher gamma activity than non-fans. This also means that the aversion to badfiction has a more decisive influence than being a fan. Only if someone is undecided about badfiction, their status as a fan becomes a decisive factor in reading behavior.

In contrast to the absolute PSD values, applying relative powers thus highlights personal variations among individuals and does not distinguish any variances between the types of texts. The comparison of the frequency bands with each other allows a more detailed analysis regarding the functionality of brain activity, which is influenced by the different habituation of the subject groups. As we are aware of the low spatial resolution of EEG data, we only roughly rely on local aspects. Our data shows that readers who read less frequently or have not engaged with the subject have higher delta band activity. Since the texts hold more unknowns for them, salience detection is particularly pronounced for these groups. Their counterparts, readers who read frequently and are more devoted to *Harry Potter* and fantasy in general, have higher theta and alpha band activities. This increased activity indicates that they read the text more attentively, reflected in greater use of short-term memory. At the same time, due to their better-trained reading skills, they require fewer mental resources for basic reading processes, such as the reception and mental structuring of the stimulus.

From a philological point of view, the different results for absolute and relative PSD values can be traced back to the very nature of badfiction as a literary meta-genre that, in its odd simplicity, undermines the involvement of readers, which is the goal of regular literature. Badfiction allows recipients to spare mental effort in a less attentive way of text processing. However, the artlessness of the genre remains a challenge for which the reading experience and reading habits make a difference. Besides reading experience in terms of familiarity (with the subject, the fantasy genre, or the literary system in general) and reading frequency in everyday life, the positive or negative attitude toward badfiction seems to influence its processing decisively.

### 4.3 Limitations of the study

The fanfiction and badfiction stimuli of this study stem from a Germanophone web forum. For comparison, a German translation of the *Harry Potter* originals was used. Since spontaneous reactions to literature and the reflexive evaluation of controversial literary artifacts both depend on cultural socialization learned through language, the deviant impact of badfiction, as found by this study, needs consolidation in other languages. First, comparing English fanfiction and badfiction with the original *Harry Potter* seems advisable.

For their attitude toward badfiction, readers were classified after the keywords they used to evaluate two randomly chosen badfiction stimuli already presented during the experiment. That this led to relatively small groups of readers who liked (*N* = 9) or disliked (*N* = 8) badfiction was certainly because most participants gave ambivalent answers. Either the two badfiction stimuli received different evaluations, one positive and the other negative, or the combination of keywords was too ambiguous to derive an apparent attitude. However, restricting the classification to unequivocal cases helped mitigate subjective interpretation. Also, it makes sense that a majority of readers has an ambivalent attitude toward such a controversial aesthetic phenomenon.

## 5 Conclusion

In this work, we investigated the influence of different variations of fantasy texts on readers, examining original *Harry Potter* texts, as well as fanfiction and badfiction about *Harry Potter*. Furthermore, we examined how various groups of participants perceive these texts differently based on their reading habits and preferences. We compared the absolute and relative EEG activities of participants while reading. Our results show that the variation of text types has a fundamentally different influence on brain activity than the readers' preferences and reading habits.

In summary, we found variations in text types to impact all readers. However, exactly how individual groups of readers respond to different texts, especially badfiction, depends on personal preferences and reading habits. We found that the different views of EEG activity, as absolute or relative powers, allow for different analyses of our data. In our results, absolute powers indicate a general, intersubjective difference in the effort required to read the text types. Relative powers indicate different distributions of brain activity across the spectrum for different groups of readers. Thus, relative powers show the characteristics of functional, subjective differences.

Based on our findings, we consider these follow-up investigations to be promising for future research: From a matrix of the pairwise coherence of every two electrodes, a network model of the connectivity between relevant brain areas could be built (Ji et al., 2018), and with locally adaptive filter algorithms (Nick et al., 2013) transformed to analyze grouping effects of text types and readers' attributes. Furthermore, we could not show how text sort and reading habits interact. Since both influences are necessarily present simultaneously in the natural act of reading, experiment designs must consider them separately. This could be achieved by a small-N design (Smith and Little, 2018) where, say, three readers of a presumably homogeneous group, like expert readers, go through large trials of badfiction and regular fiction. Together with our robust theory of what badfiction is and how it works, this would narrow down the reading habit factor and allow for a more precise inspection of text sorts, their reception, and interaction.

## Data availability statement

The datasets presented in this study can be found in online repositories. The names of the repository/repositories and accession number(s) can be found below: https://osf.io/aw9zq/?view_only=15f260ac291a4fe49000954f1f0d55d6.

## Ethics statement

The studies involving humans were approved by Darmstadt University of Technology Office of the Ethics Committee Dr. Sebastian Hartmann. The studies were conducted in accordance with the local legislation and institutional requirements. The participants provided their written informed consent to participate in this study.

## Author contributions

TW: Conceptualization, Supervision, Writing – original draft. TF: Data curation, Formal analysis, Writing – original draft. AG: Data curation, Investigation, Writing – review & editing. JB: Formal analysis, Writing – review & editing. ZP: Data curation, Investigation, Writing – review & editing.

## Footnotes

^1^The experiment was carried out by Zsofia Pilz and Anastasia Glawion as part of Pilz' master thesis (Pilz, 2023) that used Brain Vision Analyzer, the software provided by the producer of LiveAmp, for cleaning and analysis.
